# Memory, perceptual, and motor costs affect the strength of categorical encoding during motor learning of object properties

**DOI:** 10.1038/s41598-023-33515-2

**Published:** 2023-05-27

**Authors:** Evan Cesanek, J. Randall Flanagan, Daniel M. Wolpert

**Affiliations:** 1 Mortimer B. Zuckerman Mind Brain Behavior Institute, Columbia University, New York, NY USA; 2 Department of Psychology, Centre for Neuroscience Studies, Queen’s University, Kingston, ON K7L 3N6 Canada; 3 Department of Neuroscience, Columbia University, New York, NY USA

**Keywords:** Cognitive neuroscience, Human behaviour, Sensorimotor processing, Learning and memory, Motor control

## Abstract

Nearly all tasks of daily life involve skilled object manipulation, and successful manipulation requires knowledge of object dynamics. We recently developed a motor learning paradigm that reveals the categorical organization of motor memories of object dynamics. When participants repeatedly lift a constant-density “family” of cylindrical objects that vary in size, and then an outlier object with a greater density is interleaved into the sequence of lifts, they often fail to learn the weight of the outlier, persistently treating it as a family member despite repeated errors. Here we examine eight factors (Similarity, Cardinality, Frequency, History, Structure, Stochasticity, Persistence, and Time Pressure) that could influence the formation and retrieval of category representations in the outlier paradigm. In our web-based task, participants (N = 240) anticipated object weights by stretching a virtual spring attached to the top of each object. Using Bayesian *t*-tests, we analyze the relative impact of each manipulated factor on categorical encoding (strengthen, weaken, or no effect). Our results suggest that category representations of object weight are automatic, rigid, and linear and, as a consequence, the key determinant of whether an outlier is encoded as a member of the family is its discriminability from the family members.

## Introduction

Categorization is fundamental to human perception, reasoning, and communication and is viewed as a way to organize and label our experiences and support predictions in novel situations^1,2^. However, in sensorimotor control, simply labeling and making predictions about the objects we will interact with is not enough—we also have to act upon them. Indeed, the majority of tasks that we perform involve skilled interactions (e.g., lifting, stacking, pulling) with physical objects (e.g., cups, keys, scissors, zippers), which depend critically on our ability to predict their mechanical properties^3–8^. To remember such a wide range of object properties and recall them in appropriate contexts requires efficient memory organization. However, motor learning research has typically focused on tasks such as sequential button-pressing^9–11^ and goal-directed reaching^12–14^, which place minimal demands on memory organization and fail to capture the scale and structure of the world of manipulable objects. As a result, little is known about how the dynamical properties of objects are organized in memory.

In a recent study, we obtained clear evidence that memories of the motor-relevant properties of objects are organized into categories^15^. As described in detail below, when participants repeatedly lift a constant-density “family” of objects, and then a similar object with a greater density (an outlier) is introduced, they often fail to learn the weight of the outlier, persistently treating it as a family member despite repeated errors. Therefore, objects are not necessarily represented as individual items, but as part of categories that can be parameterized by common features (e.g., cups of the same density). A categorical organization would help to reduce memory requirements and computational load and facilitate generalization of learned actions to new objects.

Although we have identified a situation that leads to categorical encoding of motor-relevant object properties, we do not know the key features of this situation that lead to categorical encoding. One possibility is that the process is similar to category learning in perception and cognition, and therefore compatible with the extensive empirical data^1,2^ and well-developed theoretical models^16–19^ from these domains. Indeed, there are reasons to think that categorization in motor control may share key features identified in this literature. For instance, in our initial study^15^, we found a strong single-category focus when making sensorimotor predictions, similar to empirical findings in a reasoning task^20,21^, as well as all-or-nothing formation of new object categories, consistent with prominent computational models of category learning^17,18^. However, there are many unique aspects of motor control that may engage categorization in a different way^22^. Thus, a better understanding of categorization in the context of sensorimotor control might afford a deeper understanding of categorization in general^23^—possibly even informing future work in perception and cognition.

**Figure 1:**
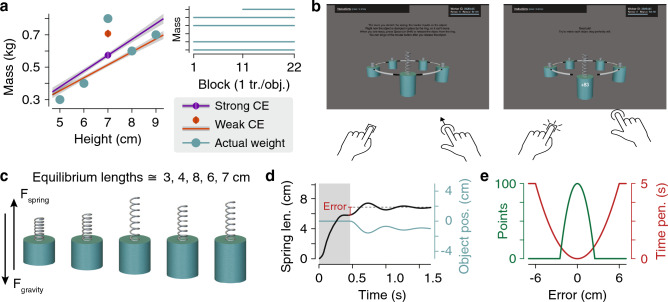
The outlier paradigm used to measure the strength of categorical encoding. (**a**) In the standard version of the task, the weights of four cylindrical objects (of equal radii) are linearly related to their volume, forming a family (first and last two blue points). The fifth, middle-size object (middle blue point) is an outlier that has a much greater density. Each object is presented once per block, except the outlier is presented only in later blocks (top-right schematic). On each trial, the participant must anticipate the weight of the presented object. After learning, the strength of categorical encoding is measured as the proximity of the anticipated weight for the outlier (purple and orange dots for two hypothetical conditions) and the anticipated weights of the family members (purple and orange regression lines for two hypothetical conditions). The hypothetical conditions show strong (purple) versus weak (orange) categorical encoding. (**b**) Example trial. The objects were clamped onto a carousel ring, which rotated to bring the target object to the front (left panel). Participants stretched a spring attached to the top of the object and pressed a button to release the clamp (right panel). The object and spring were then simulated as a mass-spring-damper to provide visual feedback about performance. Any error in the applied force would cause the object to rise or fall and oscillate. (**c**) Spring lengths that would exactly counteract the weights of the objects. (**d**) Example time series of spring length (black) and object vertical position (blue) for a trial in which the anticipated weight was less than the object weight at clamp release (end of shaded region, spring length error in red). (**e**) Points reward (green, 100 points = $0.01 bonus) and time penalty (red, added wait time before next trial) as a function of spring length error.

To demonstrate categorization in sensorimotor control, we used an ‘outlier paradigm’ (Fig. 1a). In this paradigm, participants learn to generate sensorimotor predictions of the weights of five objects (cylinders) of different sizes as they repeatedly lift them. Four of these objects—the larger two and the smaller two—share a common density (i.e*.*, their size is linearly related to their weight). In this sense, we refer to these four objects as a *family*. The fifth object, however, is not a family member—it is an *outlier*. Although its size places it in the middle of the family, it has a greater density than the family, so it weighs more than expected (based on experience with the family). The critical evidence of categorical encoding is that participants fail to improve their interactions with this outlier object, despite experiencing repeated large errors. Instead, they persistently treat it as a family member, producing anticipatory lift forces that are consistent with the learned density of the family.

Here we ask what features of the task determine the *strength* of this categorical encoding (i.e*.*, how much the outlier is attracted to the family density; see Fig. 1a for schematic of strong and weak categorical encoding). Note that although we do not present temporal learning curves, the strength of categorical encoding is inherently a measure of learning. Categorical encoding of 100% indicates a failure to learn, while lower values indicate greater learning (see Methods). We examined a variety of computationally-motivated factors that could affect the strength of categorical encoding. Though these factors were motivated from consideration of our sensorimotor task, many can be understood as extensions or adaptations of factors investigated in traditional category learning research, such as the number and diagnosticity of input dimensions^24^, the distribution of features within a category^25,26^, the separability of competing categories^24,27,28^, the presence of noise and uncertainty^20,29^, and the sequencing of stimuli^30,31^. However, other factors we manipulated show no such correspondence because they arise only in the setting of a sensorimotor task, where (i) the number of objects tends to be limited, (ii) category labels are not provided, (iii) sensitivity to within-category variability is critical to good performance, (iv) feedback is present only in the form of action outcomes, and (v) actions may also depend on movement-related costs such as execution noise, energy consumption, and time pressure. Thus, this study makes two important contributions: first, it allows us to evaluate whether category learning during object manipulation operates by the same or different principles than perceptual and cognitive category learning and, second, it serves as the first broad examination of factors specific to motor control that may affect category learning.

We used a web-based task (Fig. 1b–e) in order to recruit the large number of participants (N = 240) required to test the conditions described below. In this task, the participant views (on their computer screen) three-dimensional cylinders of varying heights and weights, which are clamped onto a circular carousel. On each trial, one object is presented at the front of the carousel and the task is to generate a lifting force that will support the weight of the object. To generate a virtual lifting force, the participant performs a click-and-drag movement to stretch a spring attached to the top of the object. The object remains stationary as the spring is stretched because it is clamped to the carousel. To release the clamp, the participant presses a keyboard button, freeing the object to move in response to gravity and the spring force they have applied (which they can no longer adjust). Any error in the applied force causes the object to rise or to fall. Participants are instructed and encouraged to stretch the spring so that each object remains perfectly still.

Although our web-based task does not require the generation of lift forces, it is certainly a motor task. As in many motor tasks, the initial conditions of the interaction are adjusted based on visual feedback, such as when aiming in archery or shooting^32^, or when lining up a putt in golf^33^. Additionally, the motor error in these examples (i.e*.,* the movement and final location of the projectile) is provided strictly through visual feedback, as in our task. Moreover, our web-based task was designed to capture the critical features of the original laboratory task, which used a robotic interface to provide haptic feedback of object weights^15^. In both versions, participants generate a targeted motor response to predict each object’s weight (either by clicking and dragging a specific distance or by generating a specific amount of force). Finally, and perhaps most importantly, we have shown in two previous studies of object lifting that the results found in the laboratory task with haptic feedback are replicated in our web-based task^15,34^.

**Figure 2:**
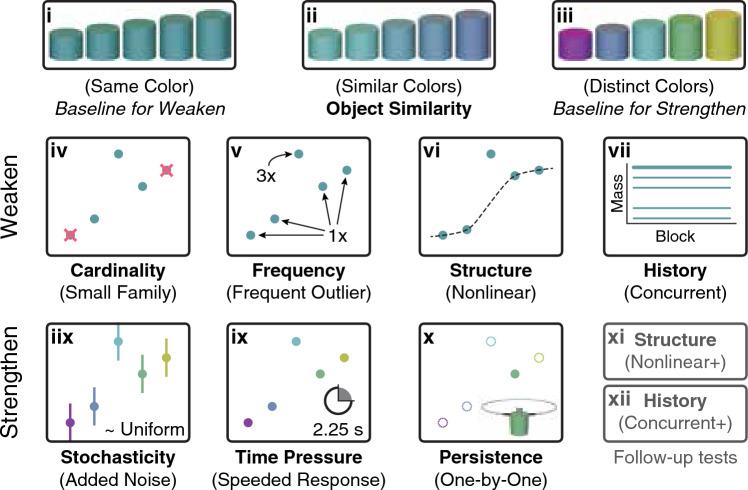
Conditions used to assess the strength of categorical encoding. Across twelve conditions, we examined the impact of eight different factors on the strength of categorical encoding (factor names in bold, condition names in parentheses). Visual similarity was manipulated in the (**i**) Same Color, (**ii**) Similar Colors, and (**iii**) Distinct Colors conditions. Same Color and Distinct Colors were then used as baseline conditions against which to compare the strength of categorical encoding under further variations. Compared to Same Color, we hypothesized weaker categorical encoding in (**iv**) Small Family: reducing cardinality of the family, (**v**) Frequent Outlier: increasing frequency of the outlier, (**vi**) Nonlinear: using a nonlinear family structure, and (**vii**) Concurrent: concurrent instead of sequential introduction of the family and outlier. Compared to Distinct Colors, we hypothesized stronger categorical encoding in (**viii**) Added Noise: adding noise to the weight feedback, (**ix**) Speeded Response: adding time pressure to stretch the spring, and (**x**) One-by-One: displaying one object at a time instead of all objects remaining visible throughout. Additionally, we ran two follow-up conditions (bottom row, gray) to determine if the Nonlinear and Concurrent conditions might strengthen categorical encoding (contrary to our initial predictions), hence the names (**xi**) Nonlinear+ and (**xii**) Concurrent+.

In the standard version of the task (Fig. 1a), there are five cylinders, four of which have a linear size-weight relationship, while the middle-size object is an outlier that weighs slightly more than the largest object. Initially, the participant interacts only with the family members in each block (one trial with each object per block) and the outlier is presented only in later blocks. Across twelve conditions, we varied a number of key factors (Fig. 2). Five factors were hypothesized to produce a weakening of categorical encoding: the visual (dis)similarity of the objects (similar or distinct colors vs. all objects the same color), the cardinality of the family (two vs. four objects), the relative frequency of interactions with the outlier to family members (3:4 vs. 1:4), the structure of the family (nonlinear vs. linear size-weight relationship), and the history of experience with the family (outlier introduced concurrently with family vs. after family). Three factors were hypothesized to produce a strengthening of categorical encoding: the amount of stochasticity (noise added to each interaction vs. no noise), the time pressure (speeded vs. unspeeded responses), and the persistence of the objects (objects presented one-by-one vs. all objects always visible). We explain the logic of these hypotheses in the Results section.

## Results

For each condition, we had a directional hypothesis about how the strength of categorical encoding might change (Fig. 2). To assess whether a particular condition affects the strength of categorical encoding, we require a reference condition that is identical in all respects except for the condition-specific manipulation. If we hypothesize a decrease in the strength of categorical encoding, we should have a reference condition with strong categorical encoding. Conversely, when we hypothesize an increase in the strength of categorical encoding, we should use a reference condition with weak categorical encoding. Based on pilot studies, we initially examined three color manipulations in order to establish conditions with strong and weak categorical encoding that would serve as these references.

### Determining reference conditions

**Figure 3:**
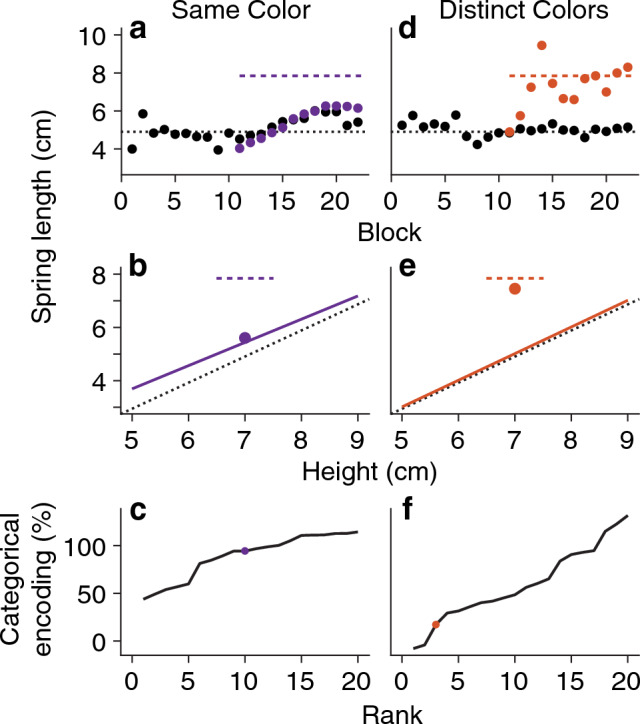
Measuring the strength of categorical encoding (reference conditions). (**a**) Timeline of average responses to the four family objects in each block (black dots) and responses to the outlier object (purple dots) for an example participant in the Same Color condition, where categorical encoding was very strong (94%). The dotted black and purple lines represent the correct average spring length for the family and correct spring length for the outlier object, respectively. (**b**) In blocks 12–22, strong categorical encoding attracts the average response to the outlier (purple dot; purple dashed line shows true weight) toward the regression line through the responses to the family objects (purple solid line). The regression line is also pulled upwards toward the true weight of the outlier (dotted black line shows the true size-weight relationship for the family). (**c**) The strength of categorical encoding for each of 20 participants in the Same Color condition, ranked in ascending order (purple dot is the example participant). (**d–f**) Same as panels a-c, but for the Distinct Colors condition, where categorical encoding was weaker. The example participant in (**e**) exhibited 17% categorical encoding.

In the Same Color condition, the cylindrical objects varied only in height, making them visually similar. Participants began by lifting the four family objects, once in each block, producing approximately correct spring forces after only a few blocks. Figure 3a shows the performance for one participant (each black point is the average force applied to the family objects in a block; black dotted line shows the average weight of the family objects). In block 11, we introduced the outlier object and this participant failed to learn the correct anticipatory force for the outlier (purple points are anticipatory forces for the outlier, which are far below the true weight shown by the dashed purple line). For this participant, the average force produced for the family was very similar to that for the outlier, suggesting strong categorical encoding.

Figure 3b shows that this participant’s average response to the outlier (purple point, average over blocks 12–22) was very close to the regression line through the responses to the family objects (purple line). Additionally, the regression line through the family objects is above the true size-weight relation for the family (black dotted line). This is another indicator of categorical encoding in the outlier paradigm: the large error for the outlier causes a small update to the category representation. Therefore, the strength of categorical encoding is indicated by the attraction of the outlier’s anticipated weight toward the family, as well as the attraction of the family regression line toward the outlier. We developed a percentage measure of categorical encoding that incorporates both factors. We take the difference between the anticipatory force for the outlier and the family-predicted weight for the outlier (e.g.*,* the distance between the purple point and the midpoint of the purple regression line) and normalize by the true difference (i.e*.*, the distance between the midpoint of the upper dashed line showing the outlier weight and the midpoint of the dotted line showing the size-weight relationship of the family). When the force for the outlier lies on the family regression line, there is 100% categorical encoding and when the outlier and family objects are learned perfectly, there is 0% categorical encoding.

Across all participants in the Same Color condition, the average strength of categorical encoding was 89.1%. Figure 3c depicts the strength of categorical encoding for individual subjects, ranked in ascending order from left to right. For most participants, we see nearly 100% categorical encoding, which means that they relied on a constant-density category representation to generate weight predictions. However, a few participants did learn the outlier to some extent. This suggests that categorical encoding in the Same Color condition was not so strong that it would entirely prevent us from observing a weakening effect in other conditions.

In the Distinct Colors condition, the family objects still shared the same density but they now had distinct colors. We hypothesized that increasing perceptual differences between objects should weaken categorical encoding. Indeed, when the outlier was introduced, many participants learned to accurately predict its weight. Figure 3d,e shows the performance of a typical participant exhibiting weak categorical encoding and Fig. 3f shows the strength of categorical encoding across all participants in ranked order. The average strength of categorical encoding was 59.5%, which constituted strong evidence that Distinct Colors led to weaker categorical encoding compared to the Same Color condition (*B*_10_ = 17, where *B* stands for Bayes factor and the subscript *10* indicates it is the ratio of the alternative hypothesis, H1, to the null, H0). However, note that approximately one-third of the participants showed very strong categorical encoding, underscoring the general idea that categorical encoding in sensorimotor tasks may arise from a combination of perceptual, memory, and motor costs that may be weighted differently across individuals.

We also examined a Similar Colors condition, in which the object colors changed gradually with size, but still fell within a single color category (“turquoise”). This made them perceptually discriminable, but there was now a structured relationship between color and size. We found that the average strength of categorical encoding was 87.7%, constituting moderate evidence for the null hypothesis that there was no change in categorical encoding strength compared to the Same Color condition (*B*_10_ = 0.25). This suggests that color variation does not necessarily reduce the strength of categorical encoding, but that imposing an arbitrary relation between size and color (e.g., Distinct Colors) does.

Based on these results, we chose to use the Same Color and Distinct Colors conditions as reference conditions that represent stronger (89.1%) and weaker (59.5%) categorical encoding. We use these as baselines to interpret categorical encoding in the remaining nine conditions.

### Factors expected to reduce categorical encoding

**Figure 4:**
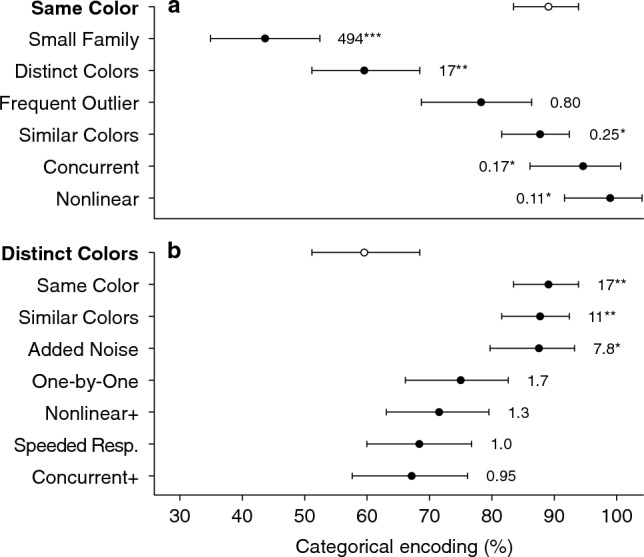
Strength of categorical encoding across conditions. (**a**) Results for all conditions that differed in only one factor from the Same Color reference condition (top row, bold font, open symbol). Data shows mean ± 1 bootstrapped SEM across participants. Bayes factors indicate relative support for a decrease in categorical encoding compared to Same Color. Comparison conditions are sorted by the magnitude of the observed effect. Asterisks indicate Bayes factor classifications (* moderate, ** strong, or *** extreme evidence). (**b**) Same as (**a**) for conditions that differed from the Distinct Colors reference condition in only one factor. Bayes factors indicate relative support for an increase in categorical encoding compared to Distinct Colors. Note that all three color conditions (Same Color, Similar Colors, Distinct Colors) appear in both panels.

Figure 4a displays the average strength of categorical encoding in each condition for which the Same Color condition can be considered a reference (i.e*.*, all conditions that differed by a single factor from Same Color). Bayes factors are displayed beside each standard error bar. Bayes factors greater than 1 represent support for a decrease in categorical encoding strength compared to the Same Color reference condition.

#### Cardinality: small family condition

Category representations are memory-efficient because they compress information about multiple instances into a reduced summary (e.g., four object weights summarized by one density). However, if one encounters only a few objects from a given category, then there is little benefit to forming a category representation. This tension motivated us to ask how the cardinality of a family impacts the strength of categorical encoding. In the Small Family condition, we found that reducing the number of objects in the family to two (by removing the smallest and largest objects) led to the weakest categorical encoding (43.7%) of all conditions, substantially reduced from the Same Color condition (*B*_10_ = 494).

#### Frequency: frequent outlier condition

In the reference condition, the outlier is experienced once for every four experiences of a family object (1:4). It may be that the outlier fails to break away from the family encoding as it is experienced so infrequently, or conversely the frequent experience of the family makes the category representation strong. Previously, we found that increasing the frequency of interactions with the outlier did not weaken categorical encoding^15^. However, this manipulation was applied only at the end of a longer experiment, after the category representation was already formed and repeatedly used, which may have influenced the results. In the Frequent Outlier condition, we re-examined this issue, tripling the frequency of interactions with the outlier from its introduction. There were now three outlier interactions to every four family interactions (3:4). Average categorical encoding strength in the Frequent Outlier condition was 78.3%, which amounted to anecdotal evidence for the null hypothesis (*B*_10_ = 0.80). Although this result is not conclusive, the absence of any change in categorical encoding would be consistent with the results of the outlier-frequency manipulation in our earlier laboratory study.

#### History: concurrent condition

Statistical models of category formation posit a “rich-get-richer” mechanism whereby the probability that a new observation belongs to a particular category in memory is proportional to the number of past observations the agent assigns to that category. Applied to the outlier paradigm, this suggests that repeatedly interacting with the family members before introducing the outlier increases the strength of categorical encoding. In the reference condition, the outlier is introduced after 40 interactions with the family members. In the Concurrent condition, the outlier was introduced from the outset, concurrently with the family members, so that all five objects were randomly interleaved from the start of the experiment. However, we found that the average strength of categorical encoding remained very high at 94.6%, providing moderate evidence in favor of the null hypothesis that there is no difference with the reference condition (*B*_10_ = 0.17; note this is a directional test so does not test for an increase in categorical encoding).

#### Structure: nonlinear condition

We also examined whether family structure has an impact on the strength of categorical encoding. Past research on function learning has suggested that humans maintain a hypothesis space with priors on different classes of function, with inductive biases toward simpler functions^35,36^. For example, the prior probability of positive-linear functions has been estimated to be approximately one order of magnitude more likely than negative-linear functions, and two to three orders of magnitude more likely than nonlinear functions. In the Random condition of our previous study^15^, we found that sets of objects with non-monotonic size-weight relationships were not learned as a category. Thus we chose a positive-sigmoidal relationship for this Nonlinear condition because it is one of the simplest nonlinear relationships. We hypothesized that a positive-sigmoidal family structure may lead to weaker categorical encoding than the positive-linear family structure of the reference condition. Contrary to this hypothesis, the nonlinear family structure did not lead to weaker categorical encoding. The average strength of categorical encoding was 99.0%, providing moderate evidence for the null hypothesis of no change in strength (*B*_10_ = 0.11).

### Factors expected to increase categorical encoding

Figure 4b displays the average strength of categorical encoding in each condition for which the Distinct Colors condition can be considered a reference (i.e*.*, all conditions that differed by a single factor from Distinct Colors). The Same Color condition is included again in this plot for comparison, though the statistical comparison with Distinct Colors was already reported above. As expected, when comparing the Similar Colors condition with Distinct Colors, we found strong evidence that categorical encoding strength was greater in Similar Colors (*B*_10_ = 11).

#### Stochasticity: added noise condition

In the Added Noise condition, we tested whether the strength of categorical encoding was affected by the amount of trial-to-trial noise in the object interactions. We hypothesized that it should be more difficult to learn to separate the outlier from the family when there is high variability in the error signals. To experimentally increase the variability, we randomly perturbed the object weight on each trial with uniformly distributed noise. The average categorical encoding strength was 87.5%, providing moderate evidence of stronger categorical encoding than the reference condition (*B*_10_ = 7.8).

#### Persistence: one-by-one condition

In the One-by-One condition, we tested the idea that uncertainty about the number of objects in a family could increase the strength of categorical encoding in order to minimize the (expected) memory cost. In the standard outlier paradigm, the objects remain visible throughout the entire experiment, so there is no uncertainty about cardinality or object identity. We hypothesized that displaying only one object at a time would increase this uncertainty, thereby increasing the strength of categorical encoding. Although the strength of categorical encoding was 75.0%, this constituted only anecdotal evidence in favor of the hypothesized increase in strength (*B*_10_ = 1.7).

#### Time pressure: speeded response condition

Our previous work showed that sensorimotor predictions are computed faster when objects are categorically encoded than when they are individually encoded^15^. However, the differences in processing that produce this advantage remain unclear. In the Speeded Response condition, we hypothesized that the strength of categorical encoding would increase when object interactions must be performed under time pressure. Participants were punished with a loss of 100 points and “time-outs” of increasing duration if they exceeded the 2.25-s time limit (approximately equivalent to the average of the median per-participant response times in other conditions, 2.33 s). This manipulation greatly sped up participants’ responses—the average of the median per-participant response times fell to 1.42 s— and participants rarely exceeded the time limit (median two trials, max eight trials). Despite the significant quickening of responses, we found that categorical encoding strength in this condition remained relatively low at 68.4%. Comparing this to the Distinct Colors condition, we obtained no evidence in favor of either the null hypothesis or the alternative hypothesis of an increase in strength (*B*_10_ = 1.0). Nonetheless, this result demonstrates that individual encoding of a distinctly colored outlier can be achieved under time pressure.

#### Follow-up experiments: nonlinear+ and concurrent+ conditions

As we found moderate support for the hypothesis that the Nonlinear and Concurrent conditions produced no change in categorical encoding relative to a decrease, we followed up by examining whether these conditions may have produced an increase in categorical encoding. Analyzing the same data but changing the specification of the alternative hypothesis, we found only anecdotal evidence of an increase in strength (Nonlinear: B_10_ = 1.5; Concurrent: B_10_ = 1.1). However, our ability to detect an increase was limited by the already-strong categorical encoding for Same Color. Thus we repeated these manipulations in two new groups with reference to the Distinct Colors condition. We refer to these conditions with distinct object colors as Nonlinear+ and Concurrent+ to indicate the altered direction of the prediction. Neither condition produced compelling evidence for an increase in categorical encoding strength compared to the Distinct Colors reference (Nonlinear+ : 71.5%, *B*_10_ = 1.3; Concurrent+ : 67.1%, *B*_10_ = 0.95). Combining the Nonlinear and Nonlinear+ conditions and comparing to a combined reference (by centering Nonlinear and Same Color categorical encoding strengths on the mean of the Same Color reference, and likewise with Nonlinear+ and Distinct Colors), we obtained stronger but still anecdotal evidence for an increase in categorical encoding (*B*_10_ = 2.2). Therefore we conclude that the Nonlinear and Concurrent manipulations did not decrease the strength of categorical encoding, while tentatively noting that a nonlinear family structure may slightly increase categorical encoding.

**Figure 5:**
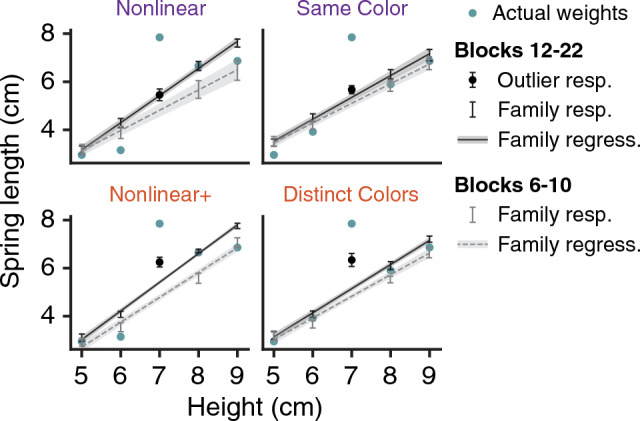
Slope of the learned size-weight mapping for the four family objects (family regression). The family regression is a linear model relating object sizes (heights) to weight predictions (spring lengths), fit separately for each participant. All data shows mean ± 1 SEM, shown by error bars or shading. The true sizes and weights of the objects are shown with blue dots. The predicted weights in blocks 12–22 are shown in black (outlier response is shown with a dot, family responses are error bars only), and the black solid line shows the family regression to these data, which was used in computing strength of categorical encoding. The gray data points and dashed regression line show the results for blocks 6–10, prior to the introduction of the outlier.

To investigate the cause of the trend in the Nonlinear conditions, we examined in detail the responses to the family objects and the regression fit. Figure 5 shows detailed results of the Nonlinear and Nonlinear+ conditions alongside the Same Color and Distinct Colors reference conditions. In the Nonlinear conditions, the average spring lengths in blocks 12–22 (black data and solid line; ignore the center black dot for the outlier) did not follow the sigmoidal shape of the family (blue dots; ignore the center blue dot for the outlier). Instead, they remained linear with respect to height: the average R^2^ of the family regression was 0.79 (95% CI = [0.73, 0.83]), similar to the average R^2^ of 0.73 (95% CI = [0.65, 0.79]) in the reference conditions. Furthermore, Fig. 5 reveals that the slopes of the family regression fits in the Nonlinear conditions (black lines, left panels) were increased by 0.21 (95% CI = [0.09, 0.32]) compared to the reference conditions (black lines, right panels). As a result, the family regressions in the Nonlinear conditions are closer to the predicted weight of the outlier (center black dot), thereby increasing our measure of categorical encoding strength (see Analysis section). The gray data and dashed lines in Fig. 5 show the responses to the family objects and the family regression fits prior to the introduction of the outlier (blocks 6–10). In the Nonlinear conditions, the responses were linear with respect to object height during these early blocks, but the regression fits do not yet show increased slopes. This implies that the slope increases were caused by experience with the heavier-than-expected outlier, which “pulled up” the linear category representation. In the reference conditions, however, we find that the slope change from the blocks before the outlier (gray) to the blocks including the outlier (black) is much smaller. In other words, the influence that the outlier exerts on the category representation seems to depend on the family structure.

## Discussion

Across 12 conditions, we evaluated how eight factors affected the strength of categorical encoding in an outlier paradigm. The factors we investigated were motivated by considering specific computational problems and pressures faced by the sensorimotor system. As in our previous work^15^, we measured the strength of categorical encoding as the degree to which the responses to the outlier and the responses to the family objects were attracted to one another. We took advantage of a web-based task in order to obtain the large sample size of 240 participants needed to test the impact of many different factors. This task has been shown to produce similar results compared to laboratory object lifting tasks in two previous studies^15,34^ and, as explained in the Introduction, it can be viewed primarily as a sensorimotor task. Nevertheless, although these previous studies suggest that similar learning processes are involved in the web-based and laboratory tasks, the fact that haptic feedback about object weight was not provided in the current experiments may limit the generality of our conclusions.

A fundamental pressure on the formation and activation of category representations is object similarity. There are two ways in which similarity could link into the likelihood of forming a new category for an outlier object. First, people likely have a prior that when objects are visually similar, they are more likely to all belong to a single category—thus biasing against new category formation. Second, the effort involved in identifying an outlier—a prerequisite for forming a new category—is greatest when the objects are more similar. While there are many ways to manipulate object appearance in the outlier paradigm, here we elected to examine the impact of the surface property of the objects, that is color, as it is believed to play a major role in memory retrieval during object recognition^37^. Differences in object color allow for stronger perceptual segregation and faster memory retrieval, possibly aided by linguistic labels. Yet the results of our color manipulations did not suggest a straightforward effect of perceptual dissimilarity. When objects were the same color, varying only in height, categorical encoding was very strong (Same Color condition) and this was unchanged when colors varied gradually and in proportion to object height (Similar Colors condition). Only in the Distinct Colors condition, where the objects were five distinct hues, did we find weaker categorical encoding. This indicates that object appearance cues that are poorly correlated with object weight are effective in weakening categorical encoding, but those that correlate with it are not. Recent work investigating the ability to learn multiple, potentially interfering sensorimotor transformations (e.g. the family and outlier) has emphasized the idea that learning is context-dependent, while also noting the difficulty of rigorously defining sensory cues that signal different contexts^38–41^. The present results suggest that to learn the outlier, the contextual cue (i.e*.*, color) should not correlate with the input of the learned transformations (i.e*.*, size).

The effectiveness of distinct colors in weakening categorical encoding may seem to be at odds with the current consensus on the status of color as a contextual cue for motor learning. Many previous studies have attempted to facilitate learning of two interfering perturbations of reaching movements (e.g., conflicting force fields or visuomotor rotations) by providing color cues, finding that they are either ineffective or require effortful explicit processing to voluntarily modify the planned movement^41–44^. In contrast, we found that many participants could use distinct colors to encode the outlier separately from the family, even when we imposed significant time pressure that reduced response times by 40% compared to unspeeded conditions. We also found no evidence of additional explicit processing in an analysis of outlier response times. Therefore our results suggest that object color can serve as an effective contextual cue for motor learning by aiding rapid selection of different category representations (i.e*.* sensorimotor transformations) when manipulating objects. This is consistent with a previous study that examined learning of weight predictions when lifting objects from two families: black cubes with a normal size-weight relationship and green cubes with an inverted size-weight relationship (though the ability to use color to select the appropriate weight prediction was impaired in an attention-demanding dual-task scenario)^45^. To reconcile these findings with the failure of color as a contextual cue in traditional motor learning paradigms, we note that the effectiveness of color cues in retrieving sensorimotor memories during object manipulation does not necessarily entail that they will also reduce interference between recently acquired, novel transformations that have strongly conflicting execution requirements (e.g., the muscle activation patterns in dual force-field learning).

We found the greatest reduction in categorical encoding in the Small Family condition, where the number of family objects was reduced to two (versus four in the Same Color reference condition). One explanation of this effect is that the relative frequency of interactions with the outlier object is doubled when there are only two instead of four family objects. However, this interpretation was not supported by our findings in the Frequent Outlier condition, where we tripled the relative frequency of the outlier but found no evidence of weaker categorical encoding. A second possibility is that the weakening effect was due to a failure to form a category representation when interacting with only two objects. Although in theory it is possible to estimate object density (a family parameter) from a single object, if we assume that the main purpose of category representations is to reduce memory load, we might expect that the memory load imposed by individual encoding (i.e*.* the number of objects) must exceed some lower limit before a category representation will be formed. Failure to form a category representation is difficult to detect, however, so the present results cannot confirm or disconfirm this hypothesis. However, we feel our results point to a third explanation, that the presence of fewer objects in the task reduces uncertainty about the identity of the outlier—in other words, it is easier to keep track of the outlier in a group of three than in a group of five. This explanation also fits with our finding that the second-largest reduction in categorical encoding was in the Distinct Colors condition, which can also be seen as reducing uncertainty about the identity of the outlier.

We also aimed to strengthen categorical encoding by increasing uncertainty about the number of objects in the family. In general, uncertainty about cardinality of a family includes the possibility that the family is countably infinite; consider the number of individual rocks, leaves, pens, or bags one could handle in a lifetime. If the formation of category representations is driven in part by computing the *expected* memory cost of a set of objects, then this uncertainty should lead to stronger category representations. Yet the One-by-One condition yielded only anecdotal evidence of increased categorical encoding compared to the Distinct Colors condition. This suggests that the expected memory load of a set of objects is not a particularly impactful factor, especially in comparison to visual distinctiveness. It is possible that the categorization mechanisms of the sensorimotor system do not take into account uncertainty about how many objects of a given type might be encountered in the future.

In the Added Noise condition, we found that categorical encoding was strengthened by adding noise to object weights. This is consistent with expected effects of noise on a learning process that involves assigning credit for error signals either to intrinsic or extrinsic sources^46–48^. Due to the inherent noise in sensorimotor processing, some error signals can be explained as the result of random fluctuations, rather than incorrect internal estimates. For instance, if an error in weight prediction is due to unpredictable variability, then it should not update the stored internal estimate of weight (or form a new category to accommodate the observation). Accordingly, we found that the strength of categorical encoding increased when trial-to-trial noise was experimentally added to the interactions. In other words, it was more difficult to learn to separate the outlier from the object family when there was high variability in the error signals. From the Added Noise condition alone, it is difficult to determine whether the noise was (a) incorporated into the category representation as a variability estimate, (b) represented independent of the category, for example being associated with the task, or (c) not represented at all, but nonetheless disrupted recognition of the outlier through feedforward processes. However, we found some evidence in favor of the first hypothesis by closely examining the Nonlinear and Nonlinear+ conditions. In Fig. 5, we showed how the family regression remained highly linear, but shifted upward via an increased slope after the introduction of the outlier. Importantly, this increase in slope does not occur in the standard version of the task with a linear size-weight relationship. This surprising result is consistent with a linear category representation that incorporates an estimate of variability. In particular, we speculate that the Nonlinear structure was (mis)interpreted as a noisy linear structure and as a result, when the outlier was introduced, the category representation was more susceptible to the “pull” of the outlier weight.

In designing the Nonlinear condition, we assumed that presenting a nonlinear family structure would provoke the formation of a nonlinear category representation, and we hypothesized this representation would lead to weaker categorical encoding due to the lower prior probability of nonlinear functions, as suggested by previous work on human function learning^35,36^. The results, however, demonstrated that our initial assumption was incorrect—participants showed a strong bias toward forming a linear category representation, despite the constant errors that resulted for some of the objects. Although the violation of our premise means that the results are not a proper test of our hypothesis, this outcome is consistent with the strong inductive bias toward linear functions indicated by the cited studies. Although Narain and colleagues^36^ concluded that participants could learn quadratic and cubic relationships between cue position and motor response timing, their results appear to be largely consistent with learning piecewise linear approximations. Future investigations should aim to establish the circumstances under which nonlinear category representations can be formed in sensorimotor tasks.

In the Concurrent and Concurrent+ conditions, the outlier was presented from the start of the experiment, instead of being introduced after a pre-training period with only the family members. This manipulation was intended to decrease the strength of categorical encoding by increasing the probability that multiple category representations would be formed, as predicted by Bayesian nonparametric models of categorization (in particular, Dirichlet process mixture models^17,19^; see Eq. 8 of the latter reference). However, we found a nominal increase in categorical encoding, inconsistent with our hypothesis. This suggests that repeated activation of a category representation during a sensorimotor task does not influence the probability that a new object category will be formed in response to error feedback. Note that this prediction comes only from a specific formulation of the Dirichlet process mixture model, in which the number of observations assigned to the category is measured in individual interactions (trials) rather than individual objects. As noted above, we were unable to either confirm or reject the hypothesis that category representations become stronger as the cardinality of the family increases, which is the main prediction of the latter version of the Dirichlet process mixture model with objects as observations. Additionally, interpretation of the inconclusive results of the Concurrent manipulation is complicated by the fact that it may also have affected the internal representation of noise. When the outlier was introduced from the start, it may have been harder to detect due to the fact that motor responses are noisier at the start of a task. As a result, the large errors with the outlier may have led to a larger estimate of category variability, which persisted throughout the task and prevented later learning of the outlier weight even after the initial motor noise had been reduced.

In the outlier paradigm, a change in our dependent variable, the strength of categorical encoding, can arise in two ways. First, from a change in how easily the outlier can be identified so that the attractive influence of the category can be suppressed, which need not involve any change in the category representation itself. Second, from a change in the strength of the category representation itself, which affects how much it constrains responses to objects such as the outlier. Although we found significant changes in the strength of categorical encoding in several conditions (Small Family, Distinct Colors, and Added Noise), these were conditions that could have affected identification of the outlier. That is, in the Small Family and Distinct Colors conditions, the outlier object is more distinctive as in the former case there are only three objects and in the latter the outlier has a different color from the family. Both these led to weaker categorical encoding. In the Added Noise condition, the outlier is less distinctive because noise tends to blur the distinction between the family and outlier, and here we found greater categorical encoding. Discounting these three conditions, the remaining task factors did not significantly impact the strength of the categorical encoding, suggesting the category representation itself is difficult to modulate. In a few conditions specifically intended to affect the strength of the category representation, we even found evidence of no change. One possible exception to this was found in our post-hoc analysis of the Nonlinear conditions, which suggested that a category representation may incorporate an estimate of variability that can affect how much it is updated in response to errors. However, this was not among the initial hypotheses of this study and therefore will require further investigation. Overall, our findings demonstrate that it is difficult to directly modulate the strength of category representations and as a result the most effective way to manipulate their influence on motor output is to facilitate (or hinder) the process of recognizing non-category members, as observed in the Small Family, Distinct Colors, and Added Noise conditions.

## Methods

### Participants

We recruited 240 participants from the Prolific online research platform (161 males, 79 females) who were between 18 and 60 years old (median 37). Participants were paid a base rate of $1.50 and received an additional bonus payment based on their final score (max bonus = $0.01/trial), resulting in an average compensation rate of $10.83 per hour (median completion time 11 min 24 s). We used the following Prolific screening criteria: located in USA, approval rate greater than 95%, age 18–60, first language English, normal or corrected-to-normal vision, not neurodivergent, not colorblind, and no diagnosis of multiple sclerosis, mild cognitive impairment/dementia, Autism Spectrum Disorder, or of any other mental illness that is uncontrolled and has a significant impact on daily life. All experiments were conducted in accordance with the 1964 Declaration of Helsinki, following protocol approved by the Columbia University Institutional Review Board. Informed consent was obtained from all participants prior to their participation.

We chose to recruit 20 participants in each condition based on a power analysis of data from two conditions of the same web-based task in our previous study. In that study, the Linear+ condition yielded strong (84%) categorical encoding, whereas the Linear++ condition yielded weaker categorical encoding (47%). For a proposed sample size *N*, we simulated 10,000 experiments. In each simulated experiment, we sampled outcomes for two groups of *N* participants from normal distributions defined using the mean and standard deviations of the Linear+ and/or Linear++ conditions. To simulate scenarios where the null hypothesis was true, we sampled both groups from the same distribution. To simulate scenarios where the alternative hypothesis was true, we sampled one group from each distribution. For each simulation, we conducted a Bayesian *t*-test as described in the current manuscript (see Analysis section). Power was calculated as the proportion of simulations that led to a Bayes factor greater than 3 or less than 1/3, as appropriate depending on the scenario being simulated. We found that a sample size of 20 yielded a power of 0.80 when testing for an increase in categorical encoding strength compared to the weaker reference condition. All other possibilities (no change in strength with both weak and strong reference conditions, and a decrease in strength compared to the strong reference condition) yielded power greater than 0.97.

### Apparatus

The experiment was created and managed using the open-source *Ouvrai* package^49^. The experiment was designed so that it could be completed only by individuals using the Google Chrome web browser in full-screen mode with pointer lock enabled on a computer with graphics hardware supporting WebGL. Mobile devices including tablets were not allowed. Participants who did not meet these requirements were blocked from starting the experiment. The task description specified that participants must use a keyboard and a mouse or trackpad to complete the task. However, the experiment could be completed using any input hardware capable of generating ‘keydown’, ‘click’, ‘mouseup’, and ‘mousemove’ DOM events in the web browser. Dimensions of the full-screen window displaying the task ranged from 1080 × 607 to 3840 × 2160 pixels. The most common dimensions were 1920 × 1080 (76 participants) and the most common aspect ratio was 1.78 (193 participants). Physical monitor sizes and viewing distances were not measured.

### Stimuli

Figure 1b shows a screenshot of the display. The objects were 3D rendered cylinders with radii of 3.5 cm and heights of 5, 6, 7, 8, and 9 cm. The on-screen height of the objects (i.e*.*, the distance between the visible vertical extrema in the image) when located at the front of the carousel ranged from 12% of the full-screen window height for the shortest object to 18.5% for the tallest object. Each object had a rendered silver metallic spring attached to its top. The objects were evenly spaced around a black and white striped ring that served as a carousel. The scene was rendered via perspective projection (70° field-of-view, aspect ratio determined by the full-screen window dimensions) to a camera 40 cm behind and 12 cm above the top of the foremost object. Feedback was provided visually via the simulated dynamics of the spring-mass-damper system. The family objects weighed 300, 400, 600, and 700 g, while the outlier object weighed 800 g. In the Sigmoidal condition, the family objects weighed 300, 320, 680, and 700 g. In the Small Family condition, only the 6-, 7-, and 8-cm cylinders were presented, which weighed 400, 800, and 600 g respectively. In the Added Noise condition, the mass of the target object was randomly perturbed by a random amount on each trial. The perturbations were drawn i.i.d. from a uniform distribution ranging from −150 to 150 g.

### Task procedure

After giving informed consent, participants were prompted to enter full screen mode and pointer lock mode by clicking labeled buttons. If they exited either of these modes during the experiment, the experiment immediately paused and prompted them to re-enter the exited mode.

On the first trial, instructions text was overlaid on the rendered 3D scene, guiding the participant through the steps of a single trial and explaining the goal of the task. These guided instructions could be repeated on the next trial by pressing the ‘d’ key at the end of the trial (until the fifth trial), or dismissed by pressing the Enter key. A collapsible panel at the top of the screen containing brief instructions could be hidden or shown at any time by pressing the ‘i’ key.

The experiment consisted of 100 trials (103 trials in the Frequent Outlier condition). During the first 40 trials, only the family objects were presented. Although the outlier was visible in the scene (except in the One-by-One condition), it was never presented as the target object (except in the Concurrent conditions, where all objects were presented from the start). The experiment was divided into blocks, where each block consisted of one trial with each of the objects. Within each block, the order of presentation of the objects was randomized, subject to the further constraint that the last object presented in block *t* could not be the first object presented in block *t* + 1 (i.e*.*, no consecutive repeats). In the Frequent Outlier condition, it was possible for the outlier to be presented in consecutive trials within a block.

### Trial procedure

Each trial consisted of two main phases: the clamp phase, in which the participant clicked and dragged vertically to stretch the spring on top of the object, and the release phase, which was triggered by pressing the spacebar (or Shift key). The release did not happen if the spring had not been stretched. The release caused the top of the spring to be fixed in space (so that it could no longer be adjusted by the participant) and the clamp section of the carousel ring around the target object to open slightly, releasing its grip on the sides of the object and the object then fell under gravity.

On release we displayed an analytical simulation of the object’s trajectory based on the initial conditions of the spring-mass-damper system (spring length set by participant, spring constant = 100, damping coefficient = 3, gravity =  − 9.81 m s^−2^). The per-trial score *y* (Fig. 1d, green) was related to the spring-length error in centimeters *e* by an inverted truncated quadratic loss function, *y* = 100*(1 − min(abs(*e*/2.5)^2^, 1), rounded to the nearest integer. The duration of the release phase in seconds *t* included a time penalty (Fig. 1e, red) that was related to the spring-length error in centimeters *e* by a truncated quadratic loss function: *t* = 1 + 5*min(abs(*e*/6)^2^, 1). The per-trial score was briefly displayed on the screen starting 100 ms into the release phase, then added to the total score displayed in a status panel in the top-right corner of the screen. During the release phase, a small loading bar in the status panel showed the time remaining until the next trial (which incorporates any time penalty).

At the end of the release phase, the object immediately returned to its initial position: clamped onto the carousel with its spring fully retracted. Following a short delay (explained below), the carousel rotated to bring the next object to the foremost position by taking the shortest angular distance. The speed of carousel rotation was 180°/s with slight in/out sinusoidal easing. As mentioned above, to ensure a consistent 1-s interval from the end of the release phase to the start of the next trial’s clamp phase, a delay of 1 s minus the duration of the upcoming carousel rotation was inserted prior to the carousel rotation.

In the One-by-One condition, the task procedure was identical, but the visibility of objects was altered. The only objects visible were a modified carousel, which did not visibly rotate and contained only one clamp region, and the current target object, which was visible only during the clamp and release phases. The carousel rotations were still executed in the experiment program to preserve timing, but no objects were visible during this time and the carousel itself remained stationary.

In the Speeded Response condition, a time limit was implemented after the first 12 trials (i.e*.,* 3 blocks). Participants were required to complete their response within 2.25 s of the start of the clamp phase (i.e*.*, when the carousel rotation stopped). This duration was selected because it was approximately equal to the average of the median per-participant response duration in unspeeded conditions. The remaining time was depicted by an animated circular icon that was wiped away in the clockwise direction on each trial. This icon was located just to the right of the target object to ensure that it was visible even without direct fixation. If the participant did not complete their response within the time limit, the object was released automatically and the trial was not repeated. However, the participant lost 100 points and was required to wait in a timeout that was initially 6 s long and increased by 2 s with each subsequent timeout. At the end of the timeout, participants had to press the Enter key to resume the experiment. These rules were designed to strongly discourage participants from letting the time run out, and in particular to discourage them from doing this repeatedly as a strategy to complete the task with minimal effort. The rules of the time limit were explained in a notification displayed when the time limit was first introduced and during any timeouts.

### Analysis

Our primary response variable was the length of the spring at the moment of the keypress to release the clamp, which corresponds to the amount of *anticipatory force* exerted on the object to support its weight. We excluded as outliers any anticipatory forces that were more than four scaled median absolute deviations from the median anticipatory force applied by a given participant to a given object, resulting in 753 exclusions (3.69%). This exclusion criterion was not applied to trials with the outlier, given that learning processes can produce large and sudden changes in responses to the outlier. Then, for each participant we measured the average anticipatory force for the family objects in block 11 onwards, and excluded any participant whose average was greater than five scaled median absolute deviations from the sample median, resulting in two exclusions. The excluded participants came from the Small Family and Frequent Outlier conditions, and accounted for 37 of the 753 individual trials that were previously excluded as outliers. Examination of the response data from these participants revealed that they gave low-effort responses. One participant repeatedly stretched the spring to its maximum length, while the other stretched it minimally. Regarding our exclusion thresholds, we aimed to be extremely conservative with individual trials and even more conservative with individual participants, thus choosing thresholds of four and five median absolute deviations based on a previous proposal that three scaled median absolute deviations is very conservative^50,51^.

For each individual, we computed a performance metric which we term *strength of categorical encoding* that measures the mutual attraction between the weight predictions for the outlier and the family. This measure is based on the difference between the predicted weight of the outlier and the weight prediction that would be expected if the outlier were a true family member (“family-predicted weight”). The predicted weight was computed as the average spring length over all outlier trials after the very first presentation (or, in the Concurrent conditions, after the tenth block). The family-predicted weight was inferred by fitting a linear regression relating object size to spring length for the four family objects (during the same trial window), then finding the predicted value corresponding to the size of the outlier. Note that because the outlier was the middle object, this value is equivalent to the average of the responses to the family objects. Finally, we normalized this difference by dividing it by the difference between the outlier’s actual weight and its weight if it were a true family member. The resulting measure is a proportion indicating the separation between the weight predictions for the family and the outlier. To obtain a percentage measure of proximity (i.e*.*, strength of categorical encoding), we subtract from one and multiply by 100.

Using this metric, we conducted Bayesian statistical analyses using the *bayesplay* package^52^ to compare specific hypotheses about the effect of each manipulated factor on the strength of categorical encoding (Table 1). The data (likelihood) model was specified as a Student’s *t-*distribution, where the mean was the mean difference in categorical encoding between conditions, the standard deviation was estimated assuming unequal variances, and the degrees of freedom were estimated using the Welch-Satterthwaite equation. The null hypothesis of no change in categorical encoding strength was specified as a point distribution at zero. The alternative hypothesis of a positive or negative effect on categorical encoding was specified as a positive or negative half-normal distribution. We chose the scale parameter of this distribution for each condition based on the corresponding reference condition. For example, when testing for a decrease in categorical encoding compared to the Same Color reference condition (in which we observed 89.1% categorical encoding), the scale parameter was set to 34.58, as this led to 99% of the probability mass falling between 0 and −89.1% categorical encoding (the theoretical minimum and maximum decrease). To compare the combined results of the Nonlinear and Nonlinear+ conditions with the combined results of the Same Color and Distinct Colors references (bottom row of Table 1), we centered the per-participant categorical encoding of each condition-reference pair on the mean of the reference condition before determining the parameters of the data model, and we set the scale parameter of the alternative hypothesis model using the mean variance of the alternative hypotheses in the separate comparisons. We report Bayes factors (*B*_10_) and interpret them according to the classification scheme suggested by Wagenmakers and colleagues^53^.

**Table 1:** Details of Bayesian statistical tests.

Reference	Condition	Mean (Δ%)	SD (Δ%)	DF	H1	H0	*B*_*10*_
Same color	Small family	−45.41	10.45	29.25	~ HN(34.58) ≤ 0	0	494.92
	Distinct colors	−29.52	10.33	31.01	~ HN(34.58) ≤ 0	0	17..33
	Frequent outlier	−10.79	10.50	29.16	~ HN(34.58) ≤ 0	0	0.80
	Similar colors	−1.34	7.59	37.97	~ HN(34.58) ≤ 0	0	0.25
	Concurrent	5.55	8.97	34.80	~ HN(34.58) ≤ 0	0	0.17
					~ HN(4.24) ≥ 0	0	1.13
	Nonlinear	9.89	8.17	37.05	~ HN(34.58) ≤ 0	0	0.11
					~ HN(4.24) ≥ 0	0	1.48
Distinct colors	Similar colors	28.18	10.41	31.54	~ HN(15.71) ≥ 0	0	10.80
	Added noise	28.00	11.18	35.62	~ HN(15.71) ≥ 0	0	7.76
	One-by-one	15.46	12.25	37.91	~ HN(15.71) ≥ 0	0	1.70
	Nonlinear+	12.00	12.19	37.87	~ HN(15.71) ≥ 0	0	1.30
	Speeded response	8.83	12.36	37.97	~ HN(15.71) ≥ 0	0	1.03
	Concurrent+	7.60	12.99	37.83	~ HN(15.71) ≥ 0	0	0.95
Same/distinct	Nonlinear/nonlinear+	10.95	7.25	78.00	~ HN(11.51) ≥ 0	0	2.16

Each row is one comparison. The data (likelihood) model was a Student’s *t* distribution with mean equal to the change in categorical encoding strength from reference to target condition, standard deviation estimated assuming unequal variances, and Welch-Satterthwaite degrees of freedom. The alternative hypothesis model was a half-normal distribution (HN) with standard deviation determined by the results of the Reference condition (see Methods). In all cases, the null hypothesis model was a point distribution at zero.

## Data Availability

All data and analysis code is available at https://www.github.com/EvanCesanek/CFW2022.
